# In conversation with Hannah Rieger

**DOI:** 10.1017/S2045796020000499

**Published:** 2020-07-07

**Authors:** Carole Tansella

**Keywords:** Art brut, contemporary art, gender differences, outsider art, women

**Fig. 1.**
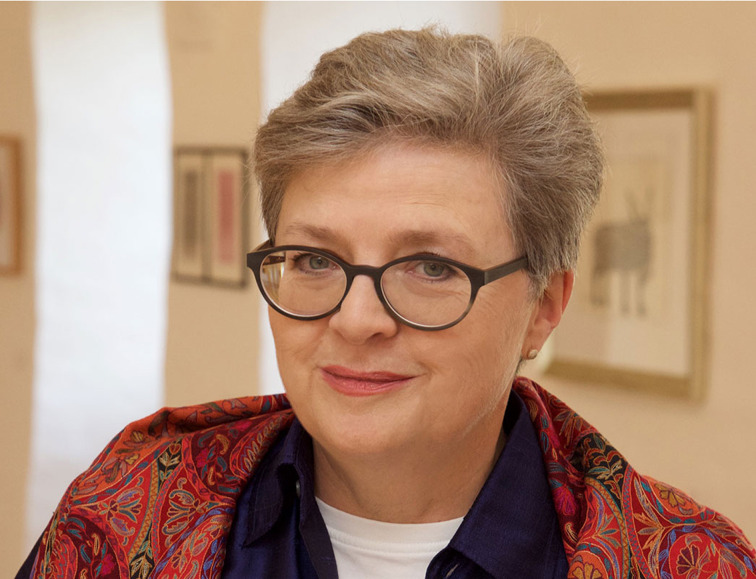
Portrait by Doris Kucera.

CT/It is with great pleasure that I welcome for this instalment of In Conversation with, the foremost European woman Art Brut collector Hannah Rieger. Based in Vienna, Hannah Rieger boasts an over 500-piece collection reflecting her interest in the art from the Austrian art studio Gugging, in female Art Brut and in contemporary non-Western Art Brut. Austria has a great history of supporting and promoting artists with disabilities. Just to remember a few, the artist Arnulf Rainer started his considerable Art Brut collection in the early Sixties; the Gugging Museum and Gallery promotes new and established talents on an international level, and the Kunst und Kultur art studio in Linz claims experience in international projects. What are your thoughts on a culture which honours the socially excluded and in which ways is your collection connected to this tradition?

**Fig. 2.**
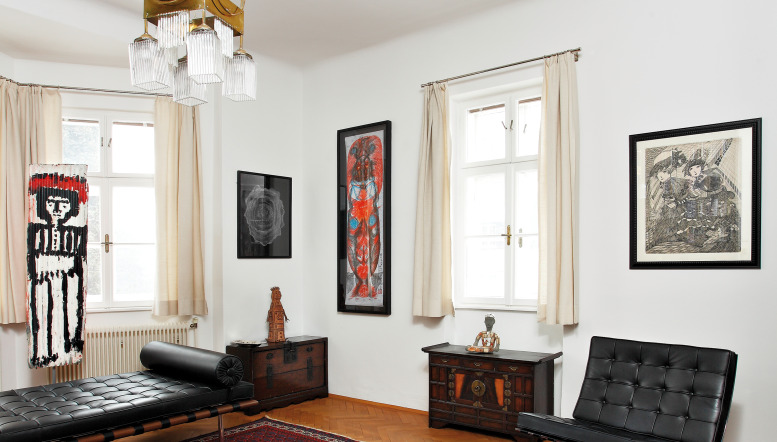
Photo by Detailsinn Fotowerkstatt.

HR/Without Sigmund Freud and his insights into the unconscious, it would probably not have been possible for the world-famous Austrian Art Brut model – which began as a men's section in a psychiatric clinic at Maria Gugging, just outside Vienna – to have evolved in this form. A culture in which the unconscious matters, and where art from the margins of society can also be linked to the unconscious, has to feel committed to the integration of its more vulnerable members. The House of Artists in Gugging, founded by Leo Navratil, was separated from the psychiatric clinic in 2007 and transformed into a modern art institution with art production (the House of Artists and the studio), a museum and a gallery. With their imaginary worlds, the artists from Gugging had the ability right from the start to create great work and make an important contribution to society. As early as 1969, Leo Navratil wrote to Jean Dubuffet and sent him some drawings, requesting an artistic assessment. Dubuffet confirmed Navratil's view of the Gugging artists' talents from an Art Brut perspective. So began the Austrian tradition of using the term Art Brut, rather than Outsider Art, and it is the term I prefer, too. For many Austrian artists, such as Arnulf Rainer, for example, Gugging has always been associated with art, in the same way, that the surrealists were amongst the first to appreciate drawings from psychiatric institutions.

The origins of my collection are profoundly linked with Gugging. My passion for Art Brut began in 1980, with an exhibition of Johann Hauser and Oswald Tschirtner at a museum in Vienna. In 1991 I began to collect. In my father's family, enthusiasm for avant-garde art was not unusual. My grandfather's brother, the dentist Heinrich Rieger, had built up an art collection during the interwar years in Vienna. He was murdered in Theresienstadt in 1942 and his collection, which also included many works by Egon Schiele, has to this day never really been reconstructed. My great-uncle was certainly a model for me as an art collector. But of course, the Holocaust – I belong to the so-called ‘second generation’, since my father survived a concentration camp – is also part of the reason for my interest in the subject of outsiders and expulsions, which has strongly influenced my identity, and my life. Symbolically, with my collection, I honour not only the Art Brut artists with their individual fates but also the members of my family who were persecuted during one of the darkest periods of our history. That is a major part of my cultural heritage too, in relation to my collection. While Art Brut is now regularly and increasingly in the spotlight in the international art world, it still does not have equal status alongside the academically recognised ‘high art’. This is particularly true for art by women. As a collector and Art Brut activist, I try to contribute to achieving this equal status.

CT/In your 2019 exhibition ‘Flying High’ (Brugger, Rieger, Rudorfer, 2019) the history of female Art Brut was displayed from its origins until today. With striking evidence, the equality of artistic power between women and men hit the visitor. Nonetheless, the romantic idea of the genius, male, artist who creates in solitude is still well alive, and it concerns both mainstream and Brut artists. It's undeniable there's a strong commercial convenience in maintaining this preconception, which became a critical habit. The art community is still pretty shy in demanding a shift from a critic and aesthetic paradigm ruled by a male-dominated point of view to an egalitarian one. What should be done to make a change happen?

HR/I think a change will happen when more and more museums, curators, gallerists, art fair organisers and collectors take up the theme of women in Art Brut. My main interest is always in the way female Art Brut artists express their regained identities – through art as an action and in the art as symbolisation.

With ‘Flying High’, which was – still hard for me to believe – the first exhibition in the world with a focus on 93 female Art Brut artists, I suddenly found myself playing a formative part in the emancipation story of women in Art Brut.

Every story of female Art Brut artists is, of course, closely related to the history of women's emancipation in general. But discrimination often manifests itself even more dramatically in the field of Art Brut. These female artists are often ‘outsiders among the outsiders’, since Art Brut still has to fight for equal status alongside the academically recognised ‘high art’.

In my life, it was my mother, a committed feminist, who particularly insisted that I should also include female artists in my collection. And this I did. For many years, men were overrepresented in my collection, because it almost exclusively featured male artists from Gugging. Laila Bachtiar remains to this day one of the rare exceptions in this Austrian Art Brut model. At some point, I realised that there are only a few independent female collectors of Art Brut in Europe. There are even fewer women who collect works by female artists.

CT/In her 1971 essay ‘Why have there been no great women artists?’ (Nochlin, 1971), American art historian Linda Nochlin reconstructs the circumstances that precluded women in the last 500 years to enter professionally the artistic career. Nochlin identifies in the internalisation of social forms of repression by women themselves, one of the reasons that prevented women to have the confidence to reach creativity heights. Today more than ever women mustn't settle for mediocrity and need to strive for excellence. From your point of view how should be acted to find, support and select women artists?

HR/Only what can be perceived exists. This is equally true for publications, exhibitions, art fairs, collections, art prizes and the secondary market. There is a huge amount of catching-up to be done in terms of support for these female artists. This is, of course, a general topic of emancipation.

With regard to finding and selecting artists, in my own experience, I have seen that the field of female Art Brut artists turns out to be much more extensive than expected by all of us. Female Art Brut has always existed but nobody paid attention to it. We have just started to raise the curtain on the wonderful female Art Brut artists in the most diverse ways. Some very encouraging developments lead me to believe that we are on the way to equal recognition for female artists. But there is still a long way to go.

At the Venice Biennale in 2013, Massimiliano Gioni's curatorial concept for the Palazzo Enciclopedico included balanced gender representation of outsider artists. Guo Fengyi and Anna Zemánková have been world-famous ever since. It is thanks to the 2017 Venice Biennale that Judith Scott is now internationally recognised. The 2019 rehang at the Museum of Modern Art in New York includes works by Judith Scott, Pearl Blauvelt and Séraphine Louis.

Aloïse Corbaz, who was the most famous of Jean Dubuffet's female artists, was often the subject of solo exhibitions – including at museum Gugging. However, the art market valuations for her work are still nowhere near the level for those by well-known male Art Brut artists.

There is, as yet, no book about Else Blankenhorn, the female star of the Prinzhorn Collection. Although Hans Prinzhorn wanted to write one himself, after the planned chapter about her in his now-famous 1922 book, ‘Artistry of the Mentally Ill’, fell victim to budgetary cuts. And despite the fact that the 2004 exhibition ‘Irre ist weiblich’ (Madness is female) at the Prinzhorn Museum in Heidelberg, a comprehensive presentation of the female artists from the Prinzhorn Collection, was highly successful. This was also my inspiration for the ‘Flying High’ exhibition in Vienna.

CT/In recent years contemporary Art Brut from Japan, India, Africa and other non-Western countries moved beyond the traditional space of local social care, gaining increasing critical and commercial attention in Europe and in the USA. ‘Flying High’ displayed several artworks by non-Western artists and part of your collecting commitment is going in this direction. How is this kind of expansion of representation of the Art Brut producers impacting the Western art market, and do you foresee any risks for this new market players to be devoured by the leading art market?

HR/I see the internationalisation of the Art Brut market as tremendously enriching. Of course, as a collector, I am delighted that Art Brut today is much more than just art from Gugging and from Austria.

Who is an artist? What is art? Such questions are subject to constant reinterpretation. Since the art market is just as exposed to the socio-economic dynamics of change as any other area of society, Art Brut is increasingly becoming a global business. The boundaries between Art Brut and ‘high art’ are shifting, partly as a result of commercial considerations. In the emancipatory drive for acceptance, Art Brut, which until recently was even more ‘raw’, authentic and subversive, is now in danger of being incorporated by the global art market. There is a very fine line between equal participation and uncritical appropriation. Do we risk losing what is special about Art Brut as it becomes commercialised?

In addition to its undeniable aesthetic impact, Art Brut must always be understood in terms of its extreme individuality. It is not constrained by the expectations of the mainstream culture and art world. It doesn't care about the competition in the academic world. Those who create these artworks often do not see themselves as artists.

This is what differentiates Art Brut from academically recognised ‘high art’, which is fundamentally influenced by the artistic and cultural mainstream and the associated discourse, as it is taught in the art universities of the world. To what extent does globally standardised art hold a mirror to our society?

I find these developments in the international art market thought-provoking. For me, it is especially problematic that as global capitalism develops it seems increasingly to absorb its most fundamental criticism – criticism by artists with a focus on emancipation and authenticity.

CT/In the last 50 years the nature of the collector as merely artist supporter has changed significantly. Today the collector is a sought-after figure for its wisdom and power. The contemporary art collector has become an active figure in the art community, shaping exhibitions, art fairs and careers. Where is collecting going today, in particular Art Brut collecting?

For some time now I have seen two parallel developments at work in the global art world in the way the awareness of Art Brut is increasing continuing specialisation on the one hand and integration with academically recognised ‘high art’ on the other. I think this is equally true for collections and for museums, galleries, art fairs and the secondary art market. On an international level, this means that more and more Art Brut is being shown publicly; not only large collections but also small, specialised ones. And in parallel, contemporary collections which have a significant focus on Art Brut are becoming more visible through exhibitions and loans. I am convinced that this parallel trend will continue in the collecting world.

Personally, I have not seen many signs that Art Brut collectors are in great demand at the moment. The same players in this field only became aware of me after I curated ‘Flying High’.

CT/Coming to the end of the interview, I won't ask you if your collection will ever be concluded: the very reason of a collection lies in its eternal missing object, we know that. Nonetheless, I would like to know what horizon you see for your collection in the next future and which challenges it will face.

HR/Not least because of the price development on the Art Brut market, I do not currently foresee any great expansion of my collection but rather at best moderate qualitative additions. I am particularly interested in works from India. In addition, of course, I support projects where I can loan or exhibit my collection.

CT/Thank you Ms Rieger for sharing your inspiring artistic vision, for guiding us into the thrilling yet often intimidating world of the art market and for showing that collecting is not just a business, it's a way of life.

## About the Author

Hannah Rieger was a manager at a specialist banking group for many years. She was also a member and Deputy Chairwoman of the University Council at the University of Applied Arts in Vienna. Today she works as a lecturer and moderator on art topics. Among her recent publications are Kunst, die verbindet (Vienna, 2014), Living in Art Brut. In total, 123 Works from the Hannah Rieger Collection (Vienna, 2017) and the exhibition catalogues Les Femmes dans l’ art brut? (Brussels, 2019), and Flying High. Female Art Brut Artists (Vienna, 2019). Hannah Rieger has been collecting Art Brut since 1991.

Carole Tansella, *Section Editor*
